# Knowledge, Attitude, and Practice Among Parents of Strabismic Children in Saudi Arabia: A Cross-Sectional Study

**DOI:** 10.7759/cureus.33120

**Published:** 2022-12-30

**Authors:** Saif Alobaisi, Azam I Alromaih, Alaa S Aljulayfi, Salam M Alanazi, Shaikha Aldossari

**Affiliations:** 1 Pediatric Ophthalmology, King Abdullah Specialist Children Hospital, Riyadh, SAU; 2 College of Medicine, King Abdulaziz Medical City, Riyadh, SAU; 3 College of Medicine, King Saud Bin Abdulaziz University for Health Sciences, Riyadh, SAU; 4 Medicine and Surgery, King Saud Bin Abdulaziz University for Health Sciences, Riyadh, SAU; 5 Ophthalmology, King Khaled Eye Specialist Hospital, Riyadh, SAU

**Keywords:** knowledge, pediatric, esotropia, exotropia, strabismus

## Abstract

Background

Strabismus is a reversible condition that must be identified and treated during the critical period of childhood. Thus, this study aims to evaluate the degree of knowledge, attitude, and practice among parents of strabismic children in Riyadh, Saudi Arabia.

Method

To this end, a cross-sectional study was conducted from August 2021 to November 2021 with a sample size of 424 parents of children with strabismus seeking ophthalmologic consultants in private and governmental ophthalmology clinics in Riyadh, Saudi Arabia. A self-administered questionnaire was used to collect the data. The questionnaire contains knowledge-related questions about strabismus, beliefs-related questions, questions addressing the barriers parents face regarding strabismus in a child, and sources of information about cross-eye and its management. Data were analysed using the SPSS database version 21 (IBM Corp., Armonk, NY, USA). P-values <0.5 was used for clinical significance.

Result

We found that most parents know there is a relationship between strabismus and refractive errors (69%) and cross eye can be corrected (55%). Moreover, strabismus causes psychosocial difficulties (55%), low self-esteem, and low school performance. Parents of strabismic children believed that their love for their children is not affected due to crossed eyes (53%) and that strabismic children should not be taken to traditional healers (59%). Barriers faced during the management of strabismus are parents’ negligence (76%), fear of surgery (34%), and high cost (29%). Doctors are the most used and preferred source of information among parents of strabismic children.

Conclusion

This study identifies gaps in knowledge, wrong beliefs in society, and the barriers faced by parents of strabismic children. Thus, raising awareness of the importance of detecting and treating strabismus early, avoiding psychosocial complications, and improving children’s quality of life.

## Introduction

The prevalence of eye disorders in children is becoming high day by day in different parts of the world. According to the WHO, over 285 million people are estimated to suffer from visual impairment worldwide [1]. Of this, 19 million are estimated to be children under the age of 14 years [1]. Globally, children living with childhood strabismus, which is a common ocular disability, have a prevalence that ranges between 1.3% and 5.7% [2]. According to the American Association for Pediatric Ophthalmology and Strabismus, an estimated 4% of children in the United States suffer from strabismus [3]. This condition might also occur at a later stage in life, thus affecting most families. The prevalence of strabismus in Saudi Arabia among children between 1 and 14 years old is 11.8% [4]. In another study conducted in Riyadh, the capital of Saudi Arabia, among 4886 strabismic patients who had surgery, the most prevalent type of strabismus was esotropia, accounting for 69.3%, whereas exotropia accounts for 26.9% [5]. In Jeddah, the most prevalent type of strabismus was esotropia, accounting for 63%, while 34.2% had exotropia and 1.4% had dissociated vertical deviation, and 82.8% of patients had bilateral strabismus [6]. In another study done in Jazan among 385 participants, the prevalence of strabismus was 36.9% [7]. In another retrospective cohort study done in the USA, the prevalence of strabismus in 10 years was 60.1% for esotropia and 32.7% for exotropia [8].

Strabismus derives from the Greek word strabismus which means “to look obliquely or to squint”. It is a disease where the eyes do not align correctly while looking at a specific object [9]. This means that one eye may gaze straight ahead to an object while the other turns downward, upward, outward, or even inward [10]. The risk factors that can lead to the development of strabismus include family history, refractive error, prematurity, and poor vision for any reason [11]. The main indication for surgical and non-surgical interventions is to restore binocular vision, correct abnormal head posture, treat double vision, and for cosmetic effect [11].

Strabismus poses social and educational challenges to children who are at risk of emotional, behavioural, and psychological difficulties, poorer social integration, and impaired self-esteem [12,13]. Most children with strabismus require developmental interventions and visual rehabilitation support. Children with strabismus increase the risk of divorce, shorter social interaction among parents, fathers working for fewer hours, or even the mother not going to work [12].

Strabismus poses a psychological impact on both children and parents, which is variable but definite and mostly depends on the economic, social, and cultural milieu [14]. Most parents seek treatment for their children based on their knowledge acquired through information technology, local physicians, and the social environment [15]. Strabismus adversely affects children’s self-image, school performance, and interpersonal relationships. Therefore, parents develop strategies to manage their children based on differing views of the social environment [15]. The parent-child relationship is adversely affected by strabismus and the child’s psychological development [16]. Furthermore, recent studies report strabismus’s growing impact, which may create a significant harmful social prejudice [14].

Strabismus is a reversible condition that must be identified and treated early. However, restoring binocularity is limited to the critical period of childhood. It is crucial to timely manage strabismus, which depends on parents’ and the population’s knowledge and attitude. Thus, this study was conducted to evaluate the degree of knowledge, attitude, and practice among parents of strabismic children in Riyadh, Saudi Arabia.

## Materials and methods

Study setting and participants

A cross-sectional study was conducted among parents of children with strabismus seeking ophthalmologic consultants in private and governmental ophthalmology clinics in Riyadh, Saudi Arabia.

The study was carried out from August 2021 to November 2021. The aim is to approximate the unknown parameters prevalence from the targeted population using random sampling. An adequate sampling size is needed to approximate the prevalence of the population having good precision, with the help of the following equation:

n = (z)2 p (1 - p) / d2

where n is the sample size, z is a statistic for 95% level of confidence which is equal to 1.96, p is the expected prevalence which is considered 0.5 with a margin of error ±5%, and d is the precision which is considered as 0.05. The sample size should be 385 parents, but to increase precision and decrease the margin of error, we increased our sample size by 10%, which resulted in a target sample size of 424. Inclusion criteria were: we included all parents between the ages of 30 and 55 who had a strabismic child and were followed up at governmental or private hospitals in Riyadh, Saudi Arabia. Exclusion criteria were parents of non-strabismic children living outside the Kingdom of Saudi Arabia. Incompletely answered questionnaires were also excluded.

Measures

This research was carried out using a self-administrated questionnaire and permission was gained from the authors to get an idea about the percentage of patients who had strabismus and did not seek medical advice. The questionnaire consists of items that can be classified into six categories. These categories contain information about the parents, information about the child with strabismus, knowledge-related questions, beliefs-related questions, barriers parents face regarding strabismus in a child, and sources of information about cross-eye and its management.

Procedures

Questionnaires were self-distributed via a convenient sampling technique by co-authors. Data were collected during four months from August 2021 to November 2021. The questionnaires were sorted based on the inclusion and exclusion criteria. Some questionnaires were removed because they were incomplete or did not meet the inclusion criteria.

Ethical considerations

The participants were given a consent form. Only co-authors had access to all parents’ replies, ensuring complete confidentiality and anonymity when gathering data and avoiding identifying information, including parents’ names. The study was approved by the Institutional Review Board. Questionnaire data were analysed using the SPSS database version 21 (IBM Corp., Armonk, NY, USA), and to confirm the null, Chi-square testing was used for statistical analysis.

## Results

A total of 424 parents responded to the questionnaire. There were 180 males, 244 females, and 12% were non-Saudi. The mean age of the enrolled parents was 36 years (SD = 9.82). The mean number of children was 3 (SD = 2), and the mean number of strabismic children was 1 (SD = 0.5). Three years old was the mean age of the child at diagnosis, and 14 years old was the maximum age at diagnosis. Most (87%) of the parents were married, 9% were divorced, and 3% were widowed. Most of the parents (65%) lived in the Centre of Saudi Arabia, 12% in the East, 8% in the North, and 7% in the South and West. Most parents (87%) were not working in the medical field; 58% of fathers had a high educational level, while 42% of the mothers had a high educational level. Most of the participating mothers completed school. The degree of deviation was mild in 39% of the children, moderate in 35%, and severe in 26%. The first question to assess parents’ knowledge was about the relationship between strabismus and refractive error; 238 (69%) respondents agreed. The knowledge assessment of the parents is described in Table 1. Most parents (55%) agreed that cross-eye could be corrected, and only 8% disagreed. Half (51%) of the parents strongly agreed that strabismus causes functional visual disability in the child, and 15% were unsure. Regarding the psychosocial difficulties of strabismus on the child, 55% strongly agreed, while 6% disagreed; 50% of the parents strongly agreed that strabismus negatively affects a child's self-esteem, and 57% strongly agreed that it negatively affects a child’s aesthetic look. Regarding how strabismus affects a child’s academic performance, 41% strongly agreed, and 10% disagreed; 31% of the parents strongly agreed that surgery for cross-eye is advised for cosmetic reasons. Moreover, 40% strongly agreed on preventing long-term vision defects by surgery; 28% of the parents agreed that eyes become crossed in a child if he/she is farsighted and does not use spectacles, 41% of the parents were unsure if a child with an abnormal head posture could have crossed eye.

**Table 1:** Knowledge-related questions

Questions	Strongly agree	
	N or Mean (SD)	Percent (%)
1. Cross-eye can be corrected	226	55
2. It causes functional visual disability to a child	207	51
3. It causes psychosocial difficulties to a child	221	55
4. It negatively affects a child’s self-esteem	204	50
5. It negatively affects a child’s aesthetic look	231	57
6. It affects a child’s school performance	167	41
7. Surgery for cross-eye is advised for cosmetic reason	126	31
8. Surgery for cross-eye prevents long-term vision defect	163	40
9. Eye becomes crossed in a child if he/she is farsighted and does not use spectacles	114	28
10. Abnormal head posture could have crossed eye in a child	40	10

Table 2 explains questions related to the belief. Starting with that parents' love for their child is affected negatively due to cross-eye, 53% strongly disagreed. On the other hand, 11% agreed; 37% of the parents had adequate knowledge regarding cross-eye; 44% of parents strongly agreed that they should take their strabismic child to an eye doctor once the child gets older. Parents equally (32%) agreed and strongly disagreed that they should consult an eye doctor as a strabismic child is a stigma to the family; 59% strongly disagreed that strabismic children should be shown to traditional healers.

**Table 2:** Belief-related questions

Questions	Strongly disagree	
	N or Mean (SD)	Percent (%)
1. Parents' love for child is affected negatively due to cross-eye	217	53
2. Parents have adequate knowledge regarding cross-eye	39	10
3. Parents should take their strabismic child to an eye doctor once the child gets older	68	17
4. Parents consult an eye doctor as strabismic child is a stigma to the family	132	32
5. Strabismic child should be shown to a traditional healer	242	59

The barriers parents face regarding strabismus in their children are reported in Table 3. A large percentage (29%) believed that parents do not treat a child with cross-eye due to high cost, and 34% do not treat their children due to fear of surgery; 33% of the parents strongly disagree that refractive error is the main reason to take the child to an eye doctor for treatment. Regarding the fear of surgery failure, 34% of parents did not seek medical help. Surprisingly, 311 parents (76%) did not care whether their child was cross-eyed, so they did not consult eye doctors.

**Table 3:** Barriers parents face regarding strabismus in a child

Questions	Agree	
	N or Mean (SD)	Percent (%)
1. Parents do not treat their child with cross-eye due to the high cost	115	29
2. Parents do not treat their child with cross-eye due to fear of surgery	137	34
3. Refractive error is the main reason to take the child to an eye doctor for treatment	91	23
4. Parents do not treat their child with cross-eye due to failure of surgery	137	34
5. Parents do not care for cross-eye in their child so do not consult eye doctors	17	4

The current source of information about cross-eye and its management is illustrated in Table 4. Doctors or healthcare workers were the current source for 79% of the responders, followed by friends and family members for 11% of the parents. The next most popular current source was social media, which had 7% of the participants, followed by search engines, which had 2%.

**Table 4:** Current sources of information about cross-eye and its management

Variables	N or Mean (SD)	Percent (%)
Doctors or healthcare workers	294	79.03
Friends / Family members	41	11.02
Social Media	28	7.53
Search engines	9	2.42

Table 5 demonstrates the preferred sources of information about cross-eye and its management. Doctors or healthcare workers were the preferred source for 86% of the responders. Friends and family members were the least preferred source with 2%. Furthermore, 3% of participants chose social media as their preferred source, while 7% chose search engines.

**Table 5:** Preferred sources of information about cross-eye and its management

Variables	N or Mean (SD)	Percent (%)
Doctors or healthcare workers	352	85.85
Friends / Family members	11	2.68
Social Media	15	3.66
Search engines	30	7.32

## Discussion

This study shows how knowledge, attitude, and practice among parents of strabismic children in Riyadh, Saudi Arabia affect early detection and treatment. Refractive errors are the second most frequently reported risk factor for developing strabismus after a family history, according to a study conducted in the Western area of Saudi Arabia [17]. Our study found that 69% of the parents were knowledgeable about the relationship between strabismus and refractive error. This could be attributed to the fact that most fathers in our study are highly educated. These findings are inconsistent with a study in Hail City which showed that only 9% knew that refractive error could cause strabismus [18]. In a study done in Riyadh among 1500 participants, only 26.2% were knowledgeable about the problems of refractive errors [19]. One study highlighted the possible complications that might occur from uncorrected refractive error [20]. These findings shed light on the importance of educating parents regarding the red flags that might lead to unpreferable complications, such as loss of stereopsis, amblyopia, and visual impairment. Moreover, there is a significant association between the level of education and the knowledge about strabismus symptoms, causes, complications, and treatment options [21]. Higher education was associated with better knowledge about strabismus. Therefore, lack of knowledge among less educated parents delayed strabismus management and increased the risk of amblyopia [14]. Other studies have found that knowledge about strabismus was poor among Ethiopian and Nigerian populations [22,23]. The differences in knowledge about strabismus could be due to different study populations and educational levels.

Strabismus treatment aims to restore binocular vision, correct double vision, and improve cosmetic appearance. Depending on the case, treatment options could include spectacles, patching of the dominant eye, or even surgery. In the current study, 55% of the parents agreed that cross-eye could be corrected, and only 8% disagreed. This study is similar to a study done in the Western Province of Saudi Arabia which found 71.5% of the population knew that strabismus could be treated [17]. On the other hand, 14.2% of the Hail population answered that glasses or contact lenses could treat strabismus, and 19% chose eye surgery [18]. The findings in this study differ from those conducted in Nigeria, where 54% in Nigeria were not familiar with strabismus treatment, and only 21% knew the medical treatment of strabismus. These findings are likely because of the lower degree of education in Nigeria, where 22% of the population are illiterate [24].

Parents’ knowledge regarding strabismus and its causes, whether it is a refractive error, amblyopia, or eye disease, plays a role in the treatment process. So, it is crucial to educate the population on the importance of early detection of strabismus through campaigns and periodic screening in schools to reduce the prevalence of strabismus and improve children’s quality of life. Not only does strabismus cause functional visual defects, but also psychosocial problems for the parent and child. More than half of parents with strabismic children in our study believed that strabismus causes psychosocial difficulties, low self-esteem, and cosmetic appearance for their children. Studies have shown that strabismus negatively affects children’s self-esteem and self-confidence [14]. Moreover, children with strabismus have difficulty making friends [14], socializing with their peers [25,26], and having lower opportunities to find jobs [14]. A study done to measure social bias against children with strabismus shows that children with strabismus were viewed negatively [25,26]. Additionally, mental health problems are greater among children with strabismus. And they are at a higher risk for anxiety, depression, and suicide [26]. Therefore, parents’ awareness of strabismus will lead to early detection and treatment, thus, avoiding psychosocial complications and improving children’s quality of life [14]. Strabismus also affects the mothers’ well-being and negatively impacts their relationship with their strabismic child. Mothers demonstrated feelings of depression, failure, hopelessness, guilt, and negativity. In the mother-child relationship, mothers were more aggressive and hostile toward their children [16].

It is important to overcome and address barriers parents face while managing their child’s strabismus. In the current study, the most common obstacle for delaying doctor consultation for strabismus is that parents do not care about their child’s crossed eyes, followed by fear of surgery and high cost. The lack of parents’ awareness of strabismus’s physical and psychosocial effects on their children could be due to parents’ ignorance. Misleading information about the complications of surgery could be a cause of surgery delay [27]. High cost was the least cited barrier, likely because the Saudi government hospitals are free to the public [28]. To address these barriers, parents’ awareness about strabismus should be increased through public education and well-rounded preoperative consultation to address patients’ concerns. Moreover, health insurance should be provided to cover the costs of strabismus surgery [27].

In the current study, the main source of patients’ knowledge was doctors or healthcare workers, friends and family members, and finally search engines. The preferred source of information about cross-eye was doctors or healthcare workers for most of the responders. Relatives and friends represented the main source of knowledge about strabismus in some studies [14,19], while in other studies the reported resources were doctors, internet browsers, and social media [29,30]. Unsurprisingly, the internet and social media use to obtain information about strabismus was the least among participants. The internet and social networks contain misconceptions and wrong information that can easily spread. On the other hand, healthcare physicians can provide care through social media platforms and not limit their face-to-face consultation to a certain time or place. Doctors share knowledge, explain procedures and their side effects, and monitor their patients. Therefore, patients make better decisions regarding the options for managing their conditions. It is important to educate patients regarding obtaining information from only healthcare physicians and correct misinformation through awareness campaigns and face-to-face consultations with doctors.

Our study highlights the importance of educating parents with low educational levels about strabismus. There are fewer studies conducted about knowledge among parents of strabismic children in Saudi Arabia. However, to the best of our knowledge, this was the first study done in Riyadh, Saudi Arabia, to assess knowledge, attitude, and practice among parents of strabismic children.

Study limitations

This study has a few limitations. First, the limitations of the current investigation are related to the inherent nature of the data collection tool which relies on the recall of the participants. Second, the study included only one region in Saudi Arabia and, hence, cannot be generalized to the whole country.

## Conclusions

Strabismus is a serious medical condition that can result in various visual issues. If treated early in life, it is a treatable ailment. Therefore, a key factor in the healing process is the parents’ knowledge and awareness of the illness. Our study demonstrated that parents of children with strabismus had a high level of understanding regarding the functional and psychological effects of strabismus on their children. Fathers’ high level of education may be responsible for the parents’ knowledge of strabismus. Surprisingly, one of the obstacles is that while having adequate knowledge about strabismus, most parents do not worry about their children having it. This can be linked to the expensive nature of medical care and the parents’ fear of surgery. Consequently, we recommend health education through campaigns and school screening programs about strabismus and its effects at the community level to prevent strabismic amblyopia and its related psychological effects if left untreated. Finally, establishing an affordable and feasible early screening program for the community is crucial.
